# Four-Year Longitudinal Epidemiological Study on the Association Between a Multi-Item Saliva Testing System and Oral and Gut Microbiota

**DOI:** 10.3390/microorganisms13112483

**Published:** 2025-10-30

**Authors:** Satoshi Sato, Daisuke Chinda, Keita Mikami, Masakazu Tobinai, Nao Ishidoya, Keisuke Furusawa, Kaede Miyashiro, Kenta Yoshida, Chikara Iino, Kaori Sawada, Tatsuya Mikami, Shigeyuki Nakaji, Koichi Murashita, Hirotake Sakuraba

**Affiliations:** 1 Department of Gastroenterology, Hematology, and Clinical Immunology, Hirosaki University Graduate School of Medicine, Hirosaki 036–8562, Japankyoshida@hirosaki-u.ac.jp (K.Y.);; 2 Division of Endoscopy, Hirosaki University Hospital, Hirosaki 036–8563, Japan; 3 Department of Preemptive Medicine, Hirosaki University Graduate School of Medicine, Hirosaki 036–8562, Japan; 4 Research Institute of Health Innovation, Hirosaki University, Hirosaki 036-8562, Japan

**Keywords:** multi-item saliva testing system, oral microbiota, gut microbiota, *Olsenella*

## Abstract

Salivary Multi Test (SMT) is a device that can quickly and noninvasively measure seven parameters related to the oral environment using saliva as a sample: (1) bacteria that cause tooth decay, (2) acidity, (3) buffering capacity, (4) occult blood, (5) white blood cells, (6) protein, and (7) ammonia. This longitudinal study aimed to investigate the relationship between SMT and oral and gut microbiota in healthy general residents. After propensity score matching, 198 participants were included (low SMT group: 99 participants; high SMT group: 99 participants). We reclassified participants four years after the follow-up survey and compared the low- and high-SMT groups. The high SMT score group indicating a poor oral environment showed increased *Olsenella* in both the oral cavity and gut at the start of the survey and four years later. Oral *Olsenella* was strongly correlated with occult blood and protein levels. In contrast, a 4-year follow-up study demonstrated that changes in oral *Olsenella* were associated with occult blood changes. Conversely, changes in gut *Olsenella* were associated with changes in occult blood and protein. A poor SMT score has been shown to be linked to increased oral and gut *Olsenella* and improving the oral environment can improve the oral–gut microbial environment.

## 1. Introduction

Deterioration of the oral environment not only leads to oral diseases such as periodontitis, dental caries, and oral cancer but also contributes to various systemic diseases, including hypertension, type 2 diabetes, atherosclerosis, non-alcoholic fatty liver disease, rheumatoid arthritis, Alzheimer’s disease, pancreatic cancer, and colorectal cancer [1,2,3]. The oral cavity is home to a microbiome second only to the gut, and oral dysbiosis causes various diseases. Migration of oral bacteria into the intestine disrupts the gut microbiota and exerts harmful effects on the body [4,5]. Particularly, when gastric or bile acids with antibacterial properties are reduced, or present in the elderly, oral bacteria can easily migrate into the intestines [6]. The relationship between oral microbiota and gut microbiota is not limited to the enteral route mentioned above but also includes hematogenous and immune cell migration routes [7]. Dental caries is associated not only with bacteria but also with oral acidity and buffering capacity [8]. Furthermore, oral occult blood, white blood cells, and proteins are important indicators of periodontal disease [9,10]. Furthermore, ammonia levels in saliva reflect oral cleanliness [11].

Saliva plays physiologically important roles in chewing, swallowing, and vocalization and contains important biological components such as enzymes and proteins, making it essential for maintaining oral homeostasis [12]. Furthermore, saliva reflects the microbiota in the gingival and subgingival microbial layers, making it a simple and noninvasive sample for evaluating oral bacterial flora [13,14]. As saliva moves into the intestines during swallowing, it is expected to influence the gut microbiota. During this process, water, lipids, and proteins in the saliva act defensively against the bactericidal effects of stomach acid, thereby promoting the migration of bacteria in the saliva to the intestines, and alter the gut microbiota [15,16,17]. Traditionally, oral environmental examinations have been conducted using methods such as culture, electrode measurements, and enzyme assays, with each parameter evaluated independently. However, to comprehensively evaluate the oral environment, a multiparameter Salivary Multi Test (SMT) using saliva as a sample, which is simple, noninvasive, and easily obtainable, was developed by Lion Corporation (Tokyo, Japan) [18,19]. The SMT system consists of test strips and a measurement device and evaluates the following parameters related to caries: (1) caries bacteria, (2) acidity, (3) buffering capacity and periodontal disease-related, (4) occult blood, (5) white blood cells, (6) protein, and oral hygiene-related (7) ammonia. SMT is characterized by its ability to perform a comprehensive, multi-item examination in a simple and short amount of time. It has been proven to be highly correlated with conventional methods such as culture, electrode, and enzyme methods, making it a useful tool for comprehensive oral examinations [18,19]. The SMT can evaluate the oral environment quickly and easily and is widely used in many dental clinics in Japan for objective explanation and health guidance. No previous studies have investigated the relationship between SMT and gut microbiota; however, since SMT serves as an indicator of periodontitis, it is suggested that SMT may be associated not only with oral bacteria but also with gut bacteria. Furthermore, although next-generation sequencers used for oral and gut microbiota analysis require significant time and cost, making them difficult to apply to routine health check-ups, SMT is quick and easy to perform, posing minimal physical or financial burden on participants.

Although SMT is a useful tool for comprehensively evaluating the oral environment, the relationship between the measured values obtained by SMT and the bacterial flora, which is an important factor in the oral environment, has not been investigated. Additionally, if SMT can be used to comprehensively evaluate the oral environment, it may have the potential to evaluate the gut microbiota.

There are many epidemiological studies on oral and gut microbiota in humans, but the results vary widely depending on the study. This is because the microbiota is influenced by numerous confounding factors, such as age, sex, body type, medication, alcohol and tobacco consumption, and history of abdominal surgery [20]. Additionally, many previous studies were cross-sectional, which may have rendered it difficult to fully adjust for confounding factors. To resolve the problems caused by the many confounding factors related to these microbiota, longitudinal analysis over a period of several years, not just cross-sectional analysis, is necessary. However, few studies have longitudinally evaluated human oral and gut microbiota. This study aimed to investigate the relationship between SMT and oral and gut microbiota in healthy residents by conducting a longitudinal study adjusted for confounding factors.

## 2. Materials and Methods

### 2.1. Study Subjects

**Figure 1:**
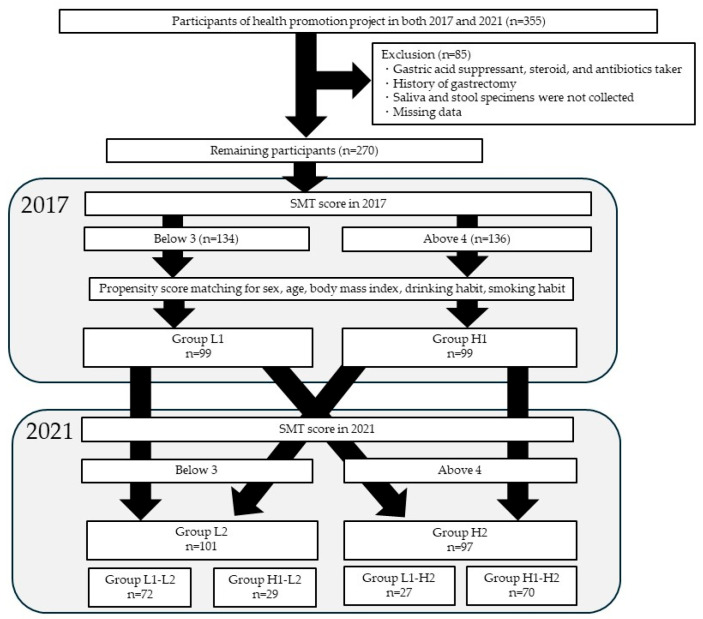
Study enrollment flowchart. SMT, Salivary Multi Test. Group L1: SMT score ≤ 3 in 2017; Group H1: SMT ≥ 4 in 2017; Group L2: SMT ≤ 3 in 2021; Group H2: SMT ≥ 4 in 2021. Group L1-L2: SMT ≤ 3 in 2017 and 2021; Group H1-L2: SMT ≥ 4 in 2017 and ≤ 3 in 2021; Group L1-H2: SMT ≤ 3 in 2017 and SMT ≥ 4 in 2021; Group H1-H2: SMT ≥ 3 in 2017 and 2021.

This study was conducted as part of the Iwaki Health Promotion Project, a community-based health promotion project targeting the general Japanese population. The Iwaki Project is an annual health checkup program for residents of the Iwaki district of Hirosaki City, Aomori Prefecture [21]. Participants were 355 adults (aged 19–93) who responded to public recruitment and participated in this project in 2017 and 2021 (Figure 1). Among these, 85 participants were excluded from the analysis if they were taking gastric acid secretion inhibitors, steroids, or antibiotics; had a history of gastric resection; did not submit saliva or stool samples; or had missing data. The remaining 270 were included in the analysis.

The median values for each item of the SMT were calculated for the 2017 and 2021 surveys. For caries, acidity, and buffering capacity, values below the median were assigned 0 points and values above the median were assigned 1 point. For occult blood, white blood cells, protein, and ammonia, values above the median were assigned 0 points and values below the median were assigned 1 point. The total score (0–7 points) for the seven items was calculated. Participants with an SMT total score of three or lower were classified into the SMT low group, and those with a score of four or higher were classified into the SMT high group. Initially, participants were categorized into the SMT low group (134 participants) and high group (136 participants) based on their SMT total scores in the 2017 survey. Furthermore, to equalize the backgrounds of both groups, 1:1 propensity score matching with a caliper width of 0.2 was performed with adjustments for sex, age, body mass index, alcohol consumption, and smoking habits, which are confounding factors that cannot be eliminated, even with exclusion criteria. As a result of propensity score matching, 198 participants, including 99 in the low SMT group (Group L1) and 99 in the high SMT group (Group H1), were identified, and a follow-up survey was conducted. Four years later, in 2021, the two groups resorted to using an SMT score of 4.0, as the cutoff value for the low SMT group (Group L2, 101 participants) and the high SMT group (Group H2, 97 participants). To further evaluate the effects of SMT on the oral and gut microbiota, participants were divided into four groups based on changes in SMT between 2017 and 2021 as follows: low-to-low SMT group (Group L1-L2, 72 participants), high-to-low group (Group H1-L2, 29 participants), low-to-high group (Group L1-H2, 27 participants), and high-to-high group (Groups H1-H2, 70 participants).

We compared the diversity and species composition of the oral and gut microbiota between the low and high SMT score groups in 2017 and 2021. The bacterial species that showed statistically significant differences between 2017 and 2021 were defined as those associated with SMT. Next, we investigated the changes in SMT parameters between 2017 and 2021 and the trends in bacterial species common to both years in the oral cavity and gut.

### 2.2. Clinical Parameters

The following parameters were investigated at the time of the surveys in 2017 and 2021: age, sex, height, weight, current and past medical histories, current medications, smoking habits, and drinking habits.

### 2.3. Salivary Multi Test (SMT)

SMT was measured according to the procedures described in the following reports [18,19]. Distilled water (3 mL) was taken into the mouth and rinsed lightly for 10 s, and the resulting sputum was used as the test sample. The test solution was dispensed onto seven test strips attached to the test paper, with 10 μL of the rinse solution applied to each strip immediately after collection. The test strips were placed in the test paper holder of the SMT measurement device. After 1 and 5 min, the test paper holder was slid to measure the color change in each test strip as reflectance (%). For cariogenic bacteria, the changes in reflectance at 1 and 5 min were measured, whereas for the other six parameters, the reflectance at 1 min was used as the measurement result. Among the seven test strips, the cariogenic bacteria test strip had a composition similar to that of the RD Test “Showa” (GC SHOWAYAKUHIN CORPORATION INC., Tokyo, Japan) The test specimens for acidity, buffering capacity, occult blood, white blood cells, and protein were similar in composition to the urine test strips “AUTION Sticks” (ARKRAY Factory, Inc., Shiga, Japan), and the test specimen for ammonia was similar in composition to “Amicheck” (ARKRAY Factory, Inc., Shiga, Japan). Caries-causing bacteria were detected by the reduction in resazurin by Gram-positive bacteria such as *Streptococcus mutans* and *Lactobacilli*. Acidity was detected by the color change in a composite pH indicator in the presence of a certain amount of acid. Occult blood was detected by the peroxidase activity of hemoglobin. White blood cells were detected by the elastase activity of white blood cells, and proteins were detected by their total protein content using a dye-binding method. Ammonia was detected based on the development of a bromocresol green color. The SMT measuring device was equipped with the same measurement mechanism as the “Pocket Chem UA PU-4010” (ARKRAY Factory, Inc., Shiga, Japan), a multi-parameter urine chemical analysis device. Specifically, the color of the test sample was measured using reflectance photometry, and the reflectance was calculated. The measurement wavelengths emitted from individual light-emitting diodes were 576, 635, and 760 nm. Higher values of cariogenic bacteria, acidity, and buffer capacity along with lower levels of occult blood, white blood cells, protein, and ammonia, were associated with a deterioration of the oral environment. A low SMT score indicates a good oral environment, while a high SMT score indicates a poor oral environment.

### 2.4. Measurements of Oral and Gut Microbiota

Data on oral and gut microbiota were obtained following the procedures described below. Participants were provided with tongue coating and stool collection kits (TechnoSuruga Laboratory Co., Ltd., Shizuoka, Japan) in advance, and tongue coating and stool samples were collected at home on the day of the study. The samples (2017) were processed using an automated nucleic acid extraction system (Precision System Science Co., Ltd., Chiba, Japan) to extract DNA from saliva and stool samples suspended using bead stirring. Magtration^®^ (Precision System Science Co., Ltd., Chiba, Japan) was used for nucleic acid extraction. The samples (2021) were processed using an automated nucleic acid extraction system GENE PREP STAR PI-480 (Kurabo Industries, Osaka, Japan) to extract DNA from saliva and stool samples suspended using bead stirring. The NR-201 reagent kit (Kurabo Industries, Osaka, Japan) was used for nucleic acid extraction. A universal primer set was used to amplify the V3-V4 region of the 16S rRNA gene. PCR amplification and solution preparation were performed as previously described [22]. PCR clean-up filter plates (Merck Millipore, Burlington, MA, USA) were used for real-time quantitative PCR quantification. The purified PCR fragments were analyzed for DNA sequencing using the MiSeq™ system (Illumina, San Diego, CA, USA) with 2 × 300-cycle paired-end sequencing. Pair-end reads were processed as follows: The adapter sequences at the 3′ end of the reads and low-quality bases (Q < 20) were trimmed using Cutadapt. Reads containing uncertain bases (N) or fewer than 150 base pairs were excluded from analysis. Pair-end reads meeting the criteria were merged into a single read called a “merged read.” The fastq_mergepairs subcommand of VSEARCH was used to exclude merged reads shorter than 370 bp or longer than 470 bp [23]. Merged reads containing multiple anticipated sequence errors were also excluded. After removing chimeric reads detected by the uchime_denovo subcommand of VSEARCH, the remaining merged reads were clustered with sequence identity ≥ 97%, and operational taxonomic units were obtained. Operational taxonomic unit classification was performed using the RDP classifier (commit hash: 701e229dde7cbe53d4261301e23459d91615999d) based on representative reads [24]. Predictions with a confidence score below 0.8 were treated as unclassified. The relative abundance of each genus in the oral and gut microbiota was calculated by dividing the number of reads per genus.

### 2.5. Statistical Analysis

Categorical variables were presented as frequencies, and continuous variables were presented as medians with interquartile ranges. Chi-square and Mann–Whitney U tests were used to compare the two groups. Changes in each SMT parameter between 2017 and 2021 were analyzed using the Wilcoxon signed-rank test. The correlation between each SMT parameter and bacterial species in 2017 and 2021 was analyzed using multiple regression analysis adjusted for confounding factors. The independent variables included sex, age, body mass index, drinking habits, and smoking habits. Prior to performing the multiple regression analysis, all continuous variables were log-transformed to approximate a normal distribution. The gut microbiota was log-transformed with a bias of +1 because some species showed 0% occupancy. The microbiota were compared using linear discriminant analysis effect size (LEfSe) [25].

Statistical analyses were performed using R software (R Foundation for Statistical Computing, version R-4.1.1) and the Statistical Package for the Social Sciences (SPSS) version 28.0 (SPSS Inc., Chicago, IL, USA). Statistical significance was set at *p* < 0.05.

### 2.6. Ethics Statement

This study was conducted in accordance with the ethical standards of the Declaration of Helsinki and was approved by the Ethics Committee of the Faculty of Medicine, Hirosaki University (approval number and date: 2021-030, approved on 4 June 2017, and 2020-046-5, approved on 1 October 2021). Written informed consent was obtained from all participants. All participants were fully informed of the purpose and procedures of the study and provided written consent.

## 3. Results

### 3.1. Participant Characteristics

The baseline characteristics of the study participants are presented in Table 1. Characteristics of Groups L1 (99 participants) and H1 (99 participants) after propensity score matching for sex, age, body mass index, alcohol consumption, and smoking habits are demonstrated in Table 2. There were no significant differences in sex, age, body mass index, alcohol consumption, or smoking habits between the two groups. The median SMT values at the time of the 2017 survey were 2.0 in Group L1 and 5.0 in Group H1. Significant differences were observed between the two groups for all seven SMT items.

### 3.2. Diversity Analysis

**Figure 2:**
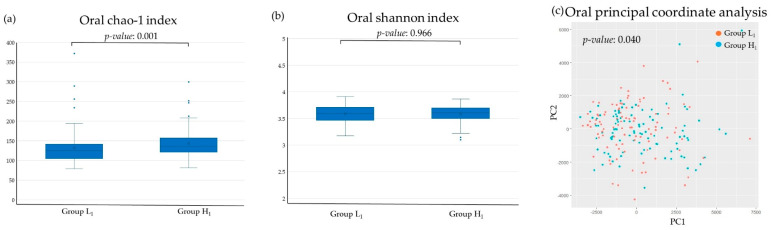
Differences in the diversity of oral microbiota in relation to the SMT: (**a**) Chao-1 index, (**b**) Shannon index, (**c**) principal coordinate analysis. Group L1: SMT score ≤ 3 in 2017. Group H1: SMT ≥ 4 in 2017. NS, not significant; pc, principal components.

**Figure 3:**
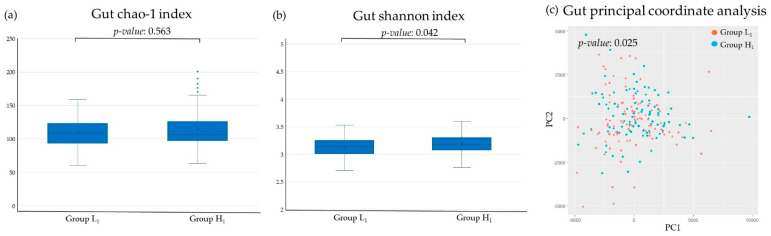
Differences in the diversity of gut microbiota in relation to the SMT: (**a**) Chao-1 index, (**b**) Shannon index, (**c**) principal coordinate analysis. Group L1: SMT score ≤ 3 in 2017. Group H1: SMT ≥ 4 in 2017. NS—not significant; pc—principal components.

Figure 2 and Figure 3 illustrate the differences in the diversity of the oral and gut micro biota. In the oral, the chao-1 index, an indicator of α diversity, was significantly higher in Group H1. While in the gut, the Shannon index, another indicator of α diversity, was higher in Group H1. Principal coordinate analysis, an indicator of β diversity, demonstrated significant differences between Group L1 and Group H1 in both the oral and gut.

### 3.3. Comparison of the SMT Score and the Oral or Gut Bacterial Species in 2017 and 2021

**Figure 4:**
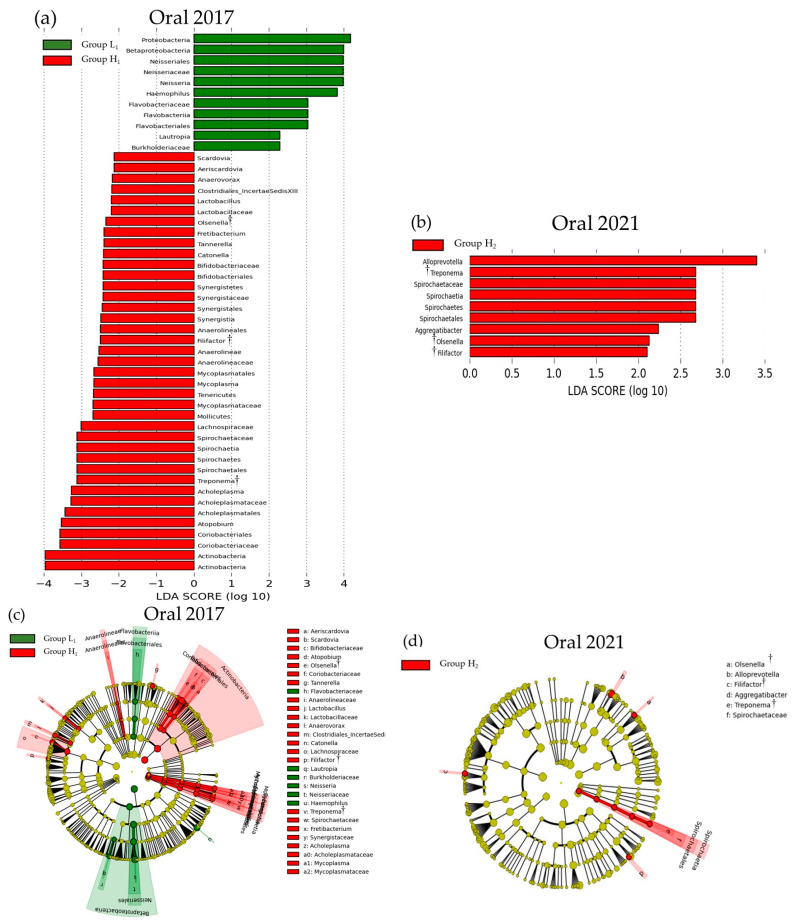
LEfSe results of the oral microbiota in the low- and high-SMT groups. (**a**) The linear discriminant for 2017. (**b**) The linear discriminant for 2021. (**c**) The cladogram report for 2017. (**d**) The cladogram report for 2021. Group L1: SMT score ≤ 3 in 2017. Group H1: SMT ≥ 4 in 2017. Group L2: SMT ≤ 3 in 2021. Group H2: SMT ≥ 4 in 2021. LDA—linear discriminant analysis. †: Bacterial species common to both 2017 and 2021.

**Figure 5:**
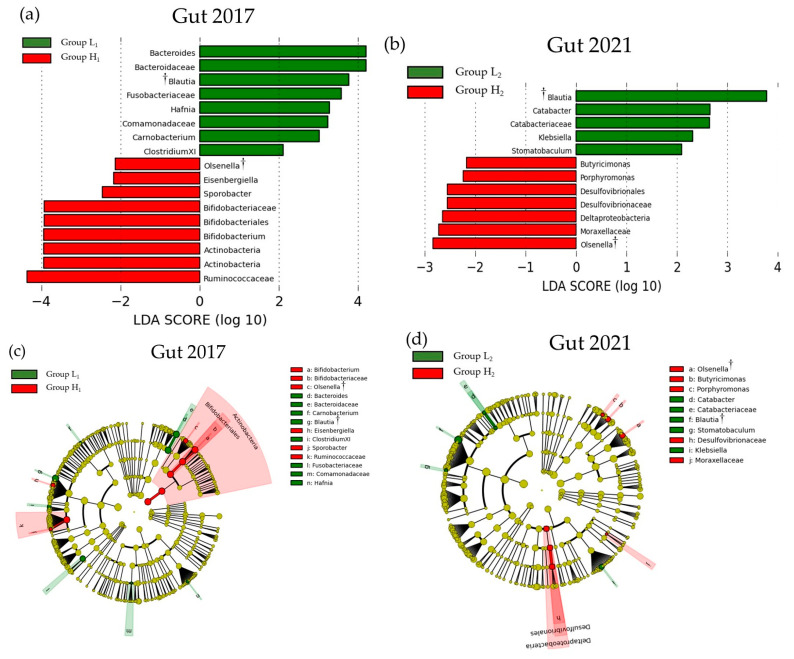
LEfSe results of the gut microbiota in the low and high SMT groups. (**a**) The linear discriminant for 2017. (**b**) The linear discriminant for 2021. (**c**) The cladogram report for 2017. (**d**) The cladogram report for 2021. Group L1: SMT score ≤ 3 in 2017. Group H1: SMT ≥ 4 in 2017. Group L2: SMT ≤ 3 in 2021. Group H2: SMT ≥ 4 in 2021. LDA—linear discriminant analysis. †: Bacterial species common to both 2017 and 2021.

Figure 4 and Figure 5 show the LEfSe results for the differences in SMT scores and oral and gut bacterial species between 2017 and 2021, respectively. In 2017, significant differences were observed between the low and high SMT score groups for 50 bacterial species in the oral cavity and 17 in the gut. In 2021, these differences were found in nine oral species and 12 gut species. In both years, the high SMT score group consistently exhibited an increase in the number of bacterial species. The bacterial genera common to both 2017 and 2021 were *Olsenella*, *Filifactor*, and *Treponema* in the oral cavity, and *Olsenella* and *Blautia* in the gut. In both 2017 and 2021, an increase in *Olsenella* was observed in the SMT high-score group in both the oral cavity and gut.

### 3.4. Correlation Between SMT and Bacterial Species

Table S1 shows the results after adjusting for confounding factors (sex, age, body mass index, drinking habits, and smoking habits) and presents the correlation between the seven SMT items and the bacterial species common to both 2017 and 2021, as identified by LEfSe. In both 2017 and 2021, occult blood, white blood cells, and protein showed significant correlations with numerous oral and gut bacteria.

### 3.5. Changes in SMT over 4 Years

Tables S2–S5 shows the changes in the SMT score and the seven parameters of the SMT between 2017 and 2021. Caries bacteria and ammonia levels significantly changed in all groups. The acidity changed significantly only in Group L1-H2. Occult blood and white blood cells changed significantly in all groups except for Groups L1-L2. In contrast, buffering capacity did not change in any of the groups.

### 3.6. Longitudinal Analysis

**Figure 6:**
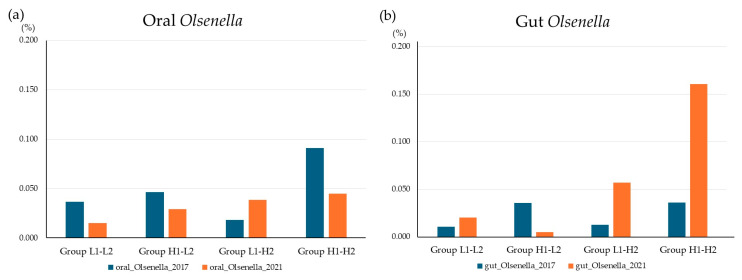
Trends in the relative abundance of oral *Olsenella* in the SMT change group between 2017 and 2021. (**a**) oral. (**b**) gut. Group L1-L2: SMT ≤ 3 in 2017 and 2021; Group H1-L2: SMT ≥ 4 in 2017 ≤ 3 in 2021; Group L1-H2: SMT ≤ 3 in 2017 and SMT ≥ 4 in 2021; Group H1-H2: SMT ≥ 3 in 2017 and 2021.

Figure 6 shows the trends in the relative abundances of oral and gut *Olsenella* in the SMT change group between 2017 and 2021. The relative abundance of *Olsenella* was generally similar in both the oral cavity and the gut. Group H1-H2 had a higher relative abundance of *Olsenella* species in both the oral cavity and gut than the other groups.

### 3.7. Olsenella Correlations

The correlations between the changes in *the Olsenella* genus and the seven SMT parameters are shown in Table 3. Changes in both oral and gut *Olsenella* were negatively correlated with changes in occult blood, whereas changes in gut *Olsenella* were negatively correlated with changes in protein levels.

## 4. Discussion

In this study, among the seven measurement items of the SMT, occult blood, white blood cells, and protein were found to be strongly associated with oral and gut microbiota compared to other items. Furthermore, the poor SMT score group showed increased *Olsenella* in both the oral and the gut, with oral *Olsenella* being particularly strongly associated with several SMT items, including acidity, occult blood, white blood cells, and protein. In contrast, a 4-year follow-up study revealed that changes in both oral and gut *Olsenella* were significantly associated with changes in occult blood.

Among the seven parameters evaluated using SMT, an increase in cariogenic bacterial count was associated with the onset and progression of periodontitis [26,27]. Patients with periodontitis have a more acidic oral environment [28,29]. Additionally, white blood cells, occult blood, and proteins are associated with periodontitis [10,30,31]. Ammonia is also an indicator of periodontal infection [32]. Thus, the SMT can comprehensively evaluate the oral environment using various indicators. In this study, the high (poor) SMT group at baseline consisted mainly of males, older individuals, and those with a high body mass index. Aging and obesity are well-known risk factors for periodontitis [33,34]. However, no sex-related differences in the prevalence of periodontitis have been reported [35]. While our study participants were primarily middle-aged and older adults, postmenopausal women have been reported to have a higher risk of periodontitis due to reduced estrogen levels, leading to decreased salivary secretion [36]. There were no significant differences in drinking or smoking habits between the low and high SMT groups; however, drinking and smoking were risk factors for periodontitis [37]. Due to these differences in the participants’ backgrounds, we performed propensity score matching with adjustments for sex, age, body mass index, alcohol consumption, and smoking habits. No previous studies have examined the effects of individual components of SMT on gut bacteria. However, factors other than caries bacteria—including acidity, buffering capacity, occult blood, white blood cells, protein, oral hygiene-related factors, and ammonia—have all been reported to be associated with periodontitis [26,27,28,29,30,31,32]. Periodontitis has an enteral route whereby oral bacteria in saliva enter the gut and cause dysbiosis. Additionally, it is known that oral bacteria can enter via a hematogenous route through mechanical injury in the oral cavity, or via an immune cell migration route by infecting immune cells [7]. Therefore, SMT is suggested to be related not only to the oral cavity but also to the gut microbiota.

In this study, the poor SMT group showed higher values of the α diversity indices Chao-1 index and Shannon index in both the oral cavity and the gut compared to the low SMT group. Previous studies have reported that patients with gingivitis or periodontitis have higher Chao-1 index values than healthy individuals [38,39]. In contrast, the Shannon index, another indicator of alpha diversity, was not found to be associated with oral environment [38]. In this study, principal coordinate analysis, an indicator of β diversity, showed significant differences between the low and high SMT score groups in both the oral and gut microbiota. Previous studies on the association between oral environment and gut microbiota diversity are limited, but there are reports suggesting that β-diversity of gut microbiota differs in patients with periodontitis [16]. The SMT-based oral evaluation suggests that it may also be useful as an indicator of gut microbiota diversity.

This study revealed that SMT was associated with both oral and gut microbiota. In particular, the genus *Olsenella* elevated in both 2017 and 2021 in the poor SMT score group, not only in the oral cavity, but also in the gut. The genus *Olsenella* consists of aerobic and anaerobic bacteria and includes six species: *Olsenella uli*, *Olsenella profusa*, *Olsenella umbonata*, *Olsenella scatoligenes*, *Olsenella timonensis*, and *Olsenella faecalis* [40]. *Olsenella* is commonly found in the oral cavity and subgingival plaques inpatients with periodontitis [41,42]. Oral *Olsenella* is not only associated with the onset and progression of apical periodontitis but has also been reported to be involved in the onset of colorectal cancer [43,44,45,46]. Additionally, *Olsenella* is present in the gut and produces short-chain fatty acids that stabilize the gut environment [47]. However, other studies have reported that gut *Olsenella* may be involved in exacerbating inflammatory responses [48]. Increased levels of gut *Olsenella* have been reported in patients with colorectal cancer, diabetic kidney disease, and juvenile idiopathic arthritis, suggesting that gut *Olsenella* may have harmful effects on the body [49,50,51]. It has been demonstrated that oral bacteria migrate to the intestine, alter the microbial flora, and consequently modify immune defense mechanisms [5,52]. This association has also been reported in patients with periodontitis [53,54]. The formation of ectopic colonies of oral bacteria in the intestine is influenced not only by the gastric acid secretion capacity and genetic susceptibility, but also by the host’s oral environment. Therefore, good oral hygiene and periodontal treatment may help to improve gut dysbiosis caused by oral bacteria [52]. In this study, it was unclear whether gut *Olsenella* originated from the oral cavity. However, this association was confirmed not only in cross-sectional but also in longitudinal studies, suggesting that the increase in gut *Olsenella* is likely a result of a deteriorated oral environment. In contrast, gut *Olsenella* showed differences from oral *Olsenella*, with no correlation with acidity or white blood cells. In this study, participants with a history of gastrectomy and those taking gastric acid secretion inhibitors, steroids, or antibiotics were excluded, and correlation calculations were adjusted for sex, age, body mass index, drinking habits, and smoking habits. However, it is likely that other factors influence the association between oral and gut microbiota. Although the design of this study cannot elucidate the mechanisms underlying the relationship between SMT and the oral and gut microbiota, the oral environment assessed by SMT may be associated not only with oral *Olsenella* but also with gut *Olsenella*.

In the present study, both oral *Filifactor* and *Treponema* increased in 2017 and 2021. Both oral *Filifactor* and *Treponema* have been reported to increase in association with periodontal disease and other oral environment deteriorations, and the results of this study support previous findings [55,56]. However, *Filifactor* and *Treponema* were not found to be associated with the gut, and the effects of both bacterial species on the intestinal environment remain unclear.

The representative oral bacteria associated with the gut include *Porphyromonas*, *Fusobacterium*, and *Streptococcus*. *Porphyromonas* is a major periodontal pathogen but is also implicated in the onset and progression of inflammatory bowel disease, diabetes, and coronary artery disease by exacerbating the Th14/Treg cell imbalance through the gut [57]. *Fusobacterium* detected in colorectal cancer tissues have been reported to originate from the oral cavity [58]. Additionally, the same urease-positive *Streptococcus* was detected in the saliva and stool samples from patients with liver cirrhosis who developed hepatic encephalopathy [59]. However, no associations were observed between SMT and these bacterial species in the present study. While previous studies focused on patients who were hospitalized or receiving outpatient care, this study targeted healthy general residents without severe underlying conditions who participated in health checkups. Owing to the significant differences in the background of the study participants, it was presumed that no association was observed with *Porphyromonas*, *Fusobacterium*, and *Streptococcus*, which are involved in relatively severe diseases.

In this study, the poor SMT group showed a decrease in gut *Blautia* in both 2017 and 2021. Additionally, gut *Blautia* was associated with several SMT measurement parameters, including caries bacteria, occult blood, white blood cells, proteins, and ammonia in 2017 and 2021, respectively. In animal experiments, oral administration of *Blautia* has been shown to increase short-chain fatty acids in the gut and reduce obesity induced by a high-fat diet [60]. Epidemiological studies have reported higher levels of the gut bacterium *Blautia* in individuals with smaller visceral fat areas [61]. Furthermore, gut *Blautia* suppresses fatty liver disease and diabetes [62,63]. Thus, gut *Blautia* is a beneficial probiotic bacterium. This study revealed that deterioration of the oral environment may harm not only the gut but also various organs throughout the body by reducing gut *Blautia*. Our study design was unable to elucidate the mechanism by which gut *Blautia* decreases with a poor SMT score, but caries bacteria, occult blood, white blood cells, proteins, and ammonia, which were found to be associated with SMT, may be involved.

This study has some limitations. First, we did not evaluate the oral hygiene status, such as periodontal pocket depth, clinical attachment level, probing bleeding, decayed, missing, filled teeth, and oral hygiene habits. Therefore, in this study, it is impossible to directly associate SMT scores with clinically defined oral diseases such as periodontitis and caries, limiting the clinical contextualization of the results. Second, participants of this study were middle-aged and older individuals living in specific to a rural Japanese area. Oral and gut microbiota vary with age and region. Therefore, the applicability of our study findings to other demographics is limited. Third, we treated the seven components of SMT equally, but occult blood, white blood cells, and protein showed strong associations with both oral and intestinal microbiota, and their respective impacts varied in magnitude. It is highly probable that the decision to assign equal weights influenced our results, which indicate that no association was observed in bacterial species other than *Olsenella*. Fourth, despite the SMT score high group having a poor oral environment, only buffering capacity showed the opposite result. A lower reflectance value for the buffer capacity indicates a stronger buffering action, which is considered protective. Buffer capacity has a smaller impact on the oral gut microbiome than the other factors; thus, it remains a topic for future research.

## 5. Conclusions

Our study revealed that the SMT score is associated with several oral and gut bacterial species, particularly *Olsenella*, which increased in both the oral and gut at the start of the survey and four years later in the poor SMT group. Furthermore, among the seven measurement items of the SMT, occult blood, white blood cells, and protein were found to be strongly associated with oral and gut microbiota compared to other items. Poor oral hygiene not only disrupts the oral microbiota but also causes dysbiosis in the gut microbiota, which is associated with the onset and progression of various systemic diseases, in addition to periodontal disease. Improving the oral environment can also improve the gut environment, leading to disease prevention and treatment, thus making oral care important for extending healthy life expectancy. Since the SMT is a simple tool for evaluating the effectiveness of oral care using only saliva samples, it is expected to have broad applicability in the future.

## Figures and Tables

**Table 1:** Participant characteristics a t baseline.

	Group L1 (*n* = 134)		Group H1 (*n* = 136)		*p*-Value
Sex, male	44 (32.8%)		64 (47.1%)		0.019
Age (years)	50.0	(39.0–59.3)	57.0	(44.3–63.0)	<0.001
Body mass index (kg/m^2^)	22.1	(19.9–24.0)	23.0	(20.5–25.3)	0.049
SMT score	2.0	(1.0–3.0)	5.0	(4.0–6.0)	<0.001
Caries bacteria	2.9	(2.5–3.5)	4.8	(4.0–6.6)	<0.001
Acidity	63.3	(54.6–72.9)	67.9	(61.4–73.4)	0.004
Buffering capacity	82.7	(75.4–88.3)	75.8	(69.5–81.2)	<0.001
Occult blood	53.7	(44.2–63.6)	29.4	(19.3–38.1)	<0.001
White blood cells	50.3	(39.5–63.2)	32.6	(29.9–37.5)	<0.001
Protein	72.5	(69.3–75.8)	61.4	(55.8–66.3)	<0.001
Ammonia	15.9	(12.5–20.1)	10.7	(9.2–12.9)	<0.001
Drinking habit	30 (22.4%)		25 (18.4%)		0.452
Smoking habit	19 (14.2%)		19 (14.0%)		0.999

Data are presented as numbers (%) or median (range). Group L1: SMT score ≤ 3 in 2017; Group H1: SMT ≥ 4 in 2017.

**Table 2:** Participants’ characteristics after matching for sex, age, and body mass index, drinking habit, and smoking habit.

	Group L1 (*n* = 99)		Group H1 (*n* = 99)		*p*-Value
Sex, male	38 (38.4%)		34 (34.3%)		0.658
Age (years)	54.0	(43.0–62.0)	54.0	(39.0–61.0)	0.695
Body mass index (kg/m^2^)	22.5	(20.1–24.4)	22.1	(19.9–24.5)	0.631
SMT score	2.0	(1.0–3.0)	5.0	(4.0–5.0)	<0.001
Caries bacteria	2.9	(2.5–3.5)	4.7	(3.8–6.6)	<0.001
Acidity	62.3	(54.5–70.5)	68.0	(62.1–73.8)	<0.001
Buffering capacity	81.9	(73.5–87.4)	77.3	(71.4–81.6)	<0.001
Occult blood	52.1	(44.2–62.9)	29.5	(17.0–38.8)	<0.001
White blood cells	49.8	(39.2–61.4)	32.7	(29.9–37.5)	<0.001
Protein	71.8	(68.8–75.4)	62.5	(56.9–67.2)	<0.001
Ammonia	14.9	(11.8–19.0)	10.9	(9.6–13.3)	<0.001
Drinking habit	27 (27.3%)		18 (18.2%)		0.174
Smoking habit	13 (13.1%)		14 (14.1%)		0.999

Data are presented as numbers (%) or median (range). Group L1: SMT score ≤ 3 in 2017; Group H1: SMT ≥ 4 in 2017.

**Table 3:** Correlation between changes in *Olsenella* genus and changes in the seven SMT parameters.

	Oral_Olsenella Change		Gut_Olsenella Change	
Change in SMT Parameters	ρ	*p*-Value	ρ	*p*-Value
Caries bacteria	0.112	0.118	−0.049	0.495
Acidity	0.103	0.149	−0.076	0.289
Buffering	−0.032	0.650	−0.119	0.094
Occult blood	−0.238	0.001	−0.207	0.003
White blood cells	−0.071	0.318	−0.087	0.224
Protein	−0.120	0.091	−0.190	0.007
Ammonia	−0.003	0.968	−0.047	0.509

ρ: Spearman’s rank correlation coefficient.

## Data Availability

The original contributions of this study are included in this article/Supplementary Materials. Further inquiries can be directed to the corresponding author.

## Supplementary Materials

The following supporting information can be downloaded at: https://www.mdpi.com/article/10.3390/microorganisms13112483/s1, Table S1: Multiple analyses between the seven SMT items and bacterial species common to both 2017 and 2021; Table S2: Changes in SMT score and seven parameters of SMT between 2017 and 2021 in GroupL1-L2; Table S3: Changes in SMT score and seven parameters of SMT between 2017 and 2021 in GroupH1-L2; Table S4: Changes in SMT score and seven parameters of SMT between 2017 and 2021 in GroupL1-H2; Table S5: Changes in SMT score and seven parameters of SMT between 2017 and 2021 in GroupH1-H2.
