# Supplementary material for: Four-Year Longitudinal Epidemiological Study on the Association Between a Multi-Item Saliva Testing System and Oral and Gut Microbiota
Source: Microorganisms. 2025 Oct 30;13(11):2483. doi: 10.3390/microorganisms13112483 (PMC12654230; doi:10.3390/microorganisms13112483)
Supplement: Supplementary file 1 [file microorganisms-13-02483-s001.zip › microorganisms-3923108-supplementary.pdf]

Table S1. Multiple analyses between the seven SMT items and bacterial species common to both 2017 and 2021.

	Caries bacteria			Acidity			Buffering capacity			Occult blood			White blood cells			Protein			Ammonia		
	β	p	R^2	β	p	R^2	β	p	R^2	β	p	R^2	β	p	R^2	β	p	R^2	β	p	R^2
2017																					
oral_*Olsenella*	0.047	0.516	0.015	0.162	0.033	0.036	-0.088	0.273	0.019	-0.357	<0.001	0.137	-0.085	0.250	0.019	-0.341	<0.001	0.119	-0.010	0.900	0.013
oral_*Filifactor*	0.155	0.030	0.049	0.188	0.013	0.057	-0.102	0.202	0.033	-0.402	<0.001	0.183	-0.267	<0.001	0.094	-0.445	<0.001	0.207	-0.034	0.661	0.026
oral_*Treponema*	0.113	0.118	0.031	0.139	0.066	0.035	-0.166	0.038	0.040	-0.442	<0.001	0.209	-0.227	0.002	0.067	-0.452	<0.001	0.206	0.009	0.914	0.018
gut_*Olsenella*	0.093	0.201	0.020	-0.008	0.912	0.012	-0.109	0.174	0.021	-0.211	0.003	0.056	-0.093	0.208	0.020	-0.206	0.006	0.051	-0.143	0.070	0.029
gut_*Blautia*	-0.151	0.036	0.042	-0.096	0.205	0.028	0.153	0.056	0.039	0.109	0.131	0.032	0.162	0.026	0.045	0.178	0.016	0.049	0.174	0.026	0.045
2021																					
oral_*Olsenella*	0.125	0.092	0.022	0.066	0.378	0.012	-0.091	0.246	0.015	-0.339	<0.001	0.119	-0.188	0.009	0.042	-0.280	<0.001	0.081	0.041	0.588	0.009
oral_*Filifactor*	0.120	0.097	0.059	0.011	0.886	0.046	-0.083	0.279	0.051	-0.318	<0.001	0.144	-0.204	0.004	0.086	-0.285	<0.001	0.121	0.095	0.198	0.054
oral_*Treponema*	0.069	0.349	0.021	-0.010	0.897	0.016	-0.097	0.212	0.024	-0.279	<0.001	0.091	-0.111	0.126	0.028	-0.302	<0.001	0.101	0.084	0.267	0.022
gut_*Olsenella*	-0.127	0.085	0.034	-0.021	0.778	0.020	-0.054	0.487	0.022	-0.104	0.155	0.029	-0.045	0.534	0.021	-0.106	0.151	0.030	-0.086	0.253	0.026
gut_*Blautia*	-0.038	0.606	0.004	0.060	0.431	0.006	0.004	0.961	0.003	0.158	0.031	0.027	0.050	0.490	0.005	0.070	0.347	0.007	0.100	0.187	0.012

Multivariate analysis was adjusted for sex, age, body mass index, alcohol habit, smoking habit. β, standardized coeffi-cient; R2, coefficient of determinatio

Table S2. Changes in SMT score and seven parameters of SMT between between 2017 and 2021 in GroupL1-L2

Group L1-L2			
	2017	2021	p-value
SMT score	2.0 (1.0-3.0)	2.0 (1.0-3.0)	0.128
Caries bacteria	3.0 (2.5-3.5)	1.2 (0.3-2.1)	<0.001
Acidity	62.7 (54.7-71.2)	66.7 (57.3-72.9)	0.285
Buffering capacity	82.4 (74.9-87.9)	82.0 (70.7-88.5)	0.511
Occult blood	56.2 (44.7-65.3)	53.8 (43.7-63.9)	0.102
White blood cells	57.0 (46.4-67.8)	57.4 (44.5-71.5)	0.413
Protein	72.9 (69.6-76.6)	72.8 (69.1-76.9)	0.512
Ammonia	15.9 (11.9-20.2)	34.1 (28.3-39.3)	<0.001

Data are presented as median (range). Group L1-L2: SMT ≤ 3 in 2017 and 2021.

Table S3. Changes in SMT score and seven parameters of SMT 2017 and 2021 in GroupH1-L2

Group H1-L2			
	2017	2021	p-value
SMT score	4.0 (4.0-5.0)	3.0 (2.0-3.0)	<0.001
Caries bacteria	4.4 (3.2-6.0)	1.9 (0.9-2.7)	<0.001
Acidity	67.6 (62.0-72.8)	66.3 (58.0-73.0)	0.215
Buffering capacity	81.0 (76.5-83.2)	74.5 (69.0-81.1)	0.056
Occult blood	38.8 (29.5-47.2)	44.3 (35.8-52.8)	0.031
White blood cells	38.3 (32.7-49.5)	52.7 (39.8-68.3)	0.001
Protein	67.5 (63.7-73.2)	71.2 (67.3-75.2)	0.008
Ammonia	11.0 (10.2-12.6)	33.9 (30.1-38.1)	<0.001

Data are presented as median (range). Group H1-L2: SMT ≥ 4 in 2017 and ≤ 3 in 2021.

Table S4. Changes in SMT score and seven parameters of SMT between 2017 and 2021 in GroupL1-H2

Group L1-H2			
	2017	2021	p-value
SMT score	3.0 (2.0-3.0)	4.0 (4.0-5.0)	<0.001
Caries bacteria	2.9 (2.6-3.5)	2.3 (1.6-3.1)	0.034
Acidity	60.6 (49.7-70.5)	66.9 (57.4-74.8)	0.009
Buffering capacity	78.5 (71.6-86.2)	74.0 (69.7-81.7)	0.394
Occult blood	45.0 (36.1-51.7)	28.3 (22.4-35.6)	<0.001
White blood cells	39.2 (33.4-44.3)	33.1 (27.7-37.4)	0.002
Protein	69.3 (64.5-72.5)	63.2 (60.5-66.0)	<0.001
Ammonia	13.6 (11.3-16.0)	29.3 (25.5-33.5)	<0.001

Data are presented as median (range). Group L1-H2: SMT ≤ 3 in 2017 and

Table S5. Changes in SMT score and seven parameters of SMT between 2017 and 2021 in GroupH1-H2

Group H1-H2			
	2017	2021	p-value
SMT score	5.0 (4.0-6.0)	5.0 (5.0-6.0)	0.667
Caries bacteria	4.8 (3.8-7.0)	2.5 (1.6-3.9)	<0.001
Acidity	68.6 (62.0-74.6)	69.7 (646.9-75.2)	0.620
Buffering capacity	75.2 (70.2-80.2)	74.0 (68.5-78.3)	0.299
Occult blood	24.0 (15.6-35.8)	20.1 (16.1-30.2)	0.018
White blood cells	31.3 (29.2-34.5)	28.8 (26.6-35.4)	0.012
Protein	60.2 (54.8-63.9)	59.8 (55.1-63.6)	0.586
Ammonia	10.8 (9.6-13.5)	28.6 (23.7-32.8)	<0.001

Data are presented as median (range). Group H1-H2: SMT ≥ 4 in 2017 and 2021. SMT ≥ 4 in 2021.
